# Bioconversion of vitamin D_3_ into calcitriol by *Actinomyces hyovaginalis* isolate CCASU- A11-2

**DOI:** 10.1186/s13568-023-01574-3

**Published:** 2023-07-12

**Authors:** Ahmad M. Abbas, Walid F. Elkhatib, Mohammad M. Aboulwafa, Nadia A. Hassouna, Khaled M. Aboshanab

**Affiliations:** 1 Department of Microbiology and Immunology, Faculty of Pharmacy, Ain Shams University, African Union Organization St. Abbassia, Cairo, 11566 Egypt; 2 Department of Microbiology & Immunology, Faculty of Pharmacy, King Salman International University, South Sinai, Egypt; 3 Department of Microbiology & Immunology, Faculty of Pharmacy, Galala University, New Galala city, Suez, Egypt

**Keywords:** *Actinomyces hyovaginalis*, Bioconversion, Calcitriol, Laboratory fermenter, Vitamin D_3_

## Abstract

Vitamin D_3_ is a fat-soluble prohormone that is activated inside the liver to produce 25-hydroxyvitamin D_3_ (calcidiol), and in the kidney to produce the fully active 1α, 25-dihydroxy vitamin D_3_ (calcitriol). A previous work piloted in our laboratory, resulted in a successful recovery of a local soil-promising *Actinomyces hyovaginalis* isolate CCASU-A11-2 capable of converting vitamin D_3_ into calcitriol. Despite the rising amount of research on vitamin D_3_ bioconversion into calcitriol, further deliberate studies on this topic can significantly contribute to the improvement of such a bioconversion process. Therefore, this work aimed to improve the bioconversion process, using the study isolate, in a 14 L laboratory fermenter (4 L fermentation medium composed of fructose (15 g/L), defatted soybean (15 g/L), NaCl (5 g/L), CaCO_3_ 2 g/L); K_2_HPO_4_, (1 g/L) NaF (0.5 g/L) and initial of pH 7.8) where different experiments were undertaken to investigate the effect of different culture conditions on the bioconversion process. Using the 14 L laboratory fermenter, the calcitriol production was increased by about 2.5-fold (32.8 µg/100 mL) to that obtained in the shake flask (12.4 µg/100 mL). The optimal bioconversion conditions were inoculum size of 2% v/v, agitation rate of 200 rpm, aeration rate of 1 vvm, initial pH of 7.8 (uncontrolled); addition of vitamin D_3_ (substrate) 48 h after the start of the main culture. In conclusion, the bioconversion of vitamin D_3_ into calcitriol in a laboratory fermenter showed a 2.5-fold increase as compared to the shake flask level where, the important factors influencing the bioconversion process were the aeration rate, inoculum size, the timing of substrate addition, and the fixed pH of the fermentation medium. So, those factors should be critically considered for the scaling-up of the biotransformation process.

**Supplementary Information:**

The online version contains supplementary material available at 10.1186/s13568-023-01574-3.

## Key points

- Bioconversion of vitamin D into calcitriol has been performed in a 14 L laboratory fermenter using a local soil-promising *Actinomyces hyovaginalis* isolate CCASU-A11-2.
- The calcitriol production was increased by about 2.5-fold (32.8 µL/100 mL) to that obtained in the shake flask (12.4 µL/100 mL).
- Aeration rate, inoculum size, the timing of substrate addition, and the fixed pH were important factors influencing the bioconversion process.

**Supplementary Information:**

The online version contains supplementary material available at 10.1186/s13568-023-01574-3.

## Introduction

Vitamin D_3_ must be activated in the liver to produce calcidiol which is hydroxylated in the renal tubules to produce the entirely active calcitriol (DeLuca 2004; Rebelos et al. 2023). The biological activities of calcitriol are required for normal physiological processes in the body including bone mineralization, muscle activity, neuronal transmission, and other important cellular activities (DeLuca 2004; Rebelos et al. 2023). Vitamin D deficiency results in the respective body functions and results in various pathological consequences such as bone-softening diseases in children and in adults (Szulc et al. 2023). Recent studies have shown a correlation between vitamin D_3_ deficiency and the occurrence of respiratory infections, cardiovascular diseases, some immunological and lung disorders, and some types of cancer (Afzal et al. 2021; Rojano-Ortega and Berral-de la Rosa 2022; Sîrbe et al. 2022; Zhang et al. 2023). The synthesis of calcitriol is compromised in patients with hepatic and/or renal problems. Accordingly, chemically synthesized calcitriol has been clinically employed in various pathological conditions, particularly renal and hepatic diseases (Grant and Holick 2005). Unfortunately, the chemical synthesis of calcitriol is a high-cost and laborious procedure that needs a lot of reaction steps (Kametani and Furuyama 1987). The microbial conversion of vitamin D_3_ has a lower cost than chemical synthesis; nevertheless, the order Actinomycetales (well known to establish vitamin D_3_ bioconversion), namely the two genera Streptomyces and *Amycolata*, showed very few microorganisms capable of conducting this conversion (Sasaki et al. 1991, 1992; Sawada et al. 2004; Kang et al. 2006; Takeda et al. 2006). Furthermore, traditional organic synthesis of the hydroxylated vitamin D3 derivatives is highly complex, which results in low yield (Andrews et al. 1986; Jin et al. 2018; Ryznar et al. 2002). Fortunately, the stereo- and regiospecific introduction of the hydroxyl group of vitamin D3 by microorganisms exhibits glowing supremacy to bypass such obstacles. In the beginning, Sasaki et al. (1991) screened about 300 *Streptomyces* isolates and found two strains with a conversion productivity of about 7.0 mg/L/min. Ehrhardt et al. (2016) used genetically engineered strains to boost the production of 25-hydroxylated vitamin D3. A study conducted in 2020 showed efficient biotransformation of vitamin D3 to 25-hydroxyvitamin D3 by a newly isolated *Bacillus cereus* strain (Tang et al. 2020). A soil isolate, *Actinomyces hyovaginalis* A11-2, was identified in a previous investigation carried out in our lab by its capacity to convert vitamin D_3_ into calcitriol (Abass et al. 2011a). This isolate was isolated from diverse soil samples taken from various locations in Egypt (Abass et al. 2011a). This was the first account of the genus *Actinomyces* converting vitamin D_3_ into its physiologically active forms. The present work aimed to study the capability of the respective isolate, to convert vitamin D_3_ into calcitriol, in a 14 L laboratory fermenter, in a batch fermentation mode. The studied culture conditions included inoculum size; agitation rate; aeration rate; initial pH; timing of substrate addition; and medium composition.

## Materials and methods

### Microorganisms and maintenance conditions

*Actinomyces hyovaginalis* A11-2 (study isolate), a local soil isolate was proven to transform vitamin D_3_ into calcitriol (Abass et al. 2011a). It was cultured onto nutrient agar slants and was incubated at 30 °C for two days then was kept at 4 °C for routine use and preserved as a stock suspension in a glycerol solution (50%) at −20 °C (Miller 1972). It was deposited in the Culture Collection Ain Shams University (CCASU) of the World Data Centre for Microorganisms (WDCM) under the code, *Actinomyces hyovaginalis* isolate CCASU-A11-2 (http://ccinfo.wdcm.org/collection/by_id/1186 (Accessed on 12 April 2023).

### Sources vitamin D_3_ and 1α,25-dihydroxy vitamin D_3_

Vitamin D_3_ was kindly provided by Medical Union Pharmaceuticals (MUP), Cairo, Egypt, while the derivative 1α,25-dihydroxy vitamin D_3_ (calcitriol) was purchased from Sigma-Aldrich, Burlington, Massachusetts,, USA.

### Basal fermentation medium

The basal medium was composed of 15 g fructose, 15 g defatted soybean, 5 g sodium chloride, 2 g calcium carbonate, 1 g dipotassium hydrogen phosphate, 0.5 g sodium fluoride per liter and initial pH of 7.8 (Abass et al. 2011b). This medium was sterilized by autoclaving at 121 °C for 15 min.

### Laboratory fermenter and its parameters

Both the growth and the capability of the tested isolate to convert vitamin D_3_ into calcitriol, were tested in a 14 L laboratory glass fermenter (CelliGen 310, Eppendorf AG, Hamburg, Germany) https://www.eppendorf.com/uploads/media/epServiceCard_BioFlo310_GB1_Ansicht_V1_25.pdf (Accessed at 12 April 2023) that provided a fully equipped system in one compact package, with its Reactor Process Control (RPC) software. The system can operate glass vessels of four sizes ranging from 0.6 L to 10 L and single-use vessels covering working volumes of 1.25 L to 40 L. This system includes a 15” industrial color touchscreen controller to regulate agitation, temperature, pH, dissolved oxygen (DO), and foam level. The reactor vessel, a 14 L vessel made of glass for visualization of the fermentation process, consisted of a stainless-steel head plate and a water-jacketed vessel body. The ports and probes were provided on the head plate. The ports are included for inoculation, acid, base, antifoam addition (polyethylene glycol, PEG 6000), a sparger, a sampling tube, and an exhaust condenser. The probes provided were a thermowell for a resistance temperature detector; a foam probe; DO probe and pH probe. The magnetic drive coupling was also located on the head plate.

A 4 L working volume of the basal fermentation medium was used for the fermentation procedure (Abass et al. 2011b). When necessary, sterile 3 N NaOH and 3 N HCl that were automatically added to maintain a consistent pH were used to manage the pH, and PEG 6000 was added to control foaming. The production medium container, antifoam (40%w/v PEG 6000), acid, and base bottles were autoclaved at 121 °C for 15 min to sterilize them. A resistance temperature detector dipped in glycerol and located in the thermowell linked to the head plate measured the temperature of the medium. A closed cooling water system provided by a chiller was used to regulate the temperature (Refrigerated Recirculator CFT-33 NESLAB Instruments, Inc., Newington, USA). A Rushton turbine impeller (six-bladed) was used for agitation and was positioned about 3 cm beneath the surface of the culture media. Air from a compressor was sent through four prefilters to remove dust, grease, and other debris, and a 0.22 m sterilizable 5 cm diameter cartridge filter sterilized the air before it entered the vessel. A ring sparger with a pressure regulator was used to introduce the sterile air into the vessel. A dissolved oxygen polarographic sensor was used to measure the dissolved oxygen percentage (DO%), and a pH probe with pH gel was used to measure the pH. (Mettler Toledo, Ohio, USA). Exhaust gases were collected from the fermenter, passed through a water-cooled condenser to eliminate moisture, and then brought back to the container. The moisture-free air was then passed through 0.22 μm exhaust filter. Before autoclaving, the pH probe was calibrated using two external buffer solutions with pH values of 7 and 4 while connected to the control unit. Once it had been sterilized, the DO probe was calibrated. Calibration was set at 0% by simply unplugging the cable, then at 100% by reconnecting the wire and running the fermenter for roughly 30 min at 400 rpm agitation rate and 4 vvm aeration rate according to the supplier’s recommendation.

### Bioconversion process

About one hundred mL Erlenmeyer flask-containing 10 mL nutrient broth was used to prepare the seed culture. A single colony of the study isolate grown onto a nutrient agar plate was picked up and used to inoculate the 10 mL of nutrient broth and then incubated for two days at 200 rpm and 30 °C. The seed culture obtained (1 × 10^5^ CFU/mL) was used to inoculate 4 L sterile basal fermentation medium, at 2% v/v and the first run ( (Run 1) was carried out under the following conditions: temperature of 30 °C, initial pH of 7.8 (uncontrolled), agitation rate of 200 rpm and aeration rate of 1 vvm. After two days of culture, 0.8 g of vitamin D_3_ dissolved in 20 mL of 96% ethanol was added as substrate. Incubation was then continued for four more days under the same conditions but at a lower temperature of 28 °C. At the completion of the batch fermentation cycle, a 50 ml aliquot was analyzed for vitamin D_3_ and calcitriol, and the viable count technique was used to estimate the growth of the study isolate and all experiments were done in triplicate.

### Effect of different culture conditions on the fermentation process

Different culture conditions that may affect vitamin D_3_ bioconversion by the study isolate were studied. The results obtained were compared with those achieved in the basic run (Run 1), which was used as a control. In any optimization run, the examined factor value that showed to be the best one for calcitriol production was applied as in the ones that followed.

#### Studied factors included

1. Aeration rates of 0.1 vvm and 2 vvm; one at a time.
2. Agitation rate of 400 rpm.
3. Inoculum size of 4% v/v.
4. Using skim milk, as the nitrogen source, instead of soybean.
5. Using glucose, as the carbon source, instead of fructose.
6. Adding substrate three days after the beginning of the main culture.
7. Fixing pH at 7.8 (controlled), simultaneously with substrate addition.

The list of the different conditions of the nine fermentation runs (run-1-run 9) is displayed in  Additional file 1: Table S1.

### Extraction and preparation of residual vitamin D_3_ and its metabolites for analysis

After the completion of each fermentation run, residual vitamin D_3_ and its metabolites were extracted according to the method described by Bligh and Dyer (1995), with minor modifications Abass et al. 2011a).

### Preparation of growth supernatant

A total of 50 mL Fermentation liquor was collected in a plastic falcon tube and centrifuged at 4000 rpm for 20 min at 4 °C then the growth supernatant was collected in 250 mL Erlenmeyer flask.

### Extraction of vitamin D_3_ and its metabolites

The resulting supernatant was shaken at room temperature at 140 rpm for 40 min after being combined with 50 ml of the methanol/methylene chloride mixture (2:1). Following the addition of 25 mL of methylene chloride, the mixture was shaken for an additional 40 min. Once transferred to a separating funnel, the entire contents of the 250 mL flask were left to stand until complete phase separation. The leftover aqueous layer was similarly re-extracted as previously indicated but with half quantities of the organic solvents used before. The lower clear organic layer (primary extract) was collected. After phase separation, the secondary extract (organic layer) was gathered and mixed with the primary extract in an appropriate evaporating flask.

### The concentration of the organic extract

The collected organic extract obtained was evaporated, under vacuum, using a rotary evaporator at 40 °C. The dried residue obtained was dissolved in 1.5 mL methanol of HPLC grade (vitamin D_3_ bioconversion concentrated extract).

### Analysis by high performance liquid chromatography (HPLC)

During this experiment (Run 1) and all subsequent ones, HPLC technique was used to quantify the production of calcitriol by the study isolate, based on the obtained HPLC peak area. An aliquot (20 µL) of concentrated vitamin D_3_ bioconversion extract was examined by HPLC under the following circumstances: A stationary phase consisting of a Discovery/Supelco HS C18 column with an inner diameter of 4.6 mm and a length of 25 cm, a mobile phase consisting of acetic acid (0.1 v/v in water) and acetonitrile (55:45), and a UV-VIS detector with a wavelength of 254 nm. HPLC analysis was first performed for a mixture of methanolic solutions of calcitriol standard, vitamin D_3_, and 1-hydroxy vitamin D_3_ (used as an analogue for rough detection of 25-hydroxyvitamin D_3_ since it was commercially unavailable) to determine the retention time and standardize the analysis. Following that, calcitriol concentrations in all samples were assessed via a calibration curve created using a calcitriol standard analyzed by HPLC under identical conditions.

### Statistical analysis

Statistical analysis displayed as mean ± S.D was done using the Excel Microsoft Office 365.

## Results

### Study isolate growth and bioconversion process

**Fig. 1:**
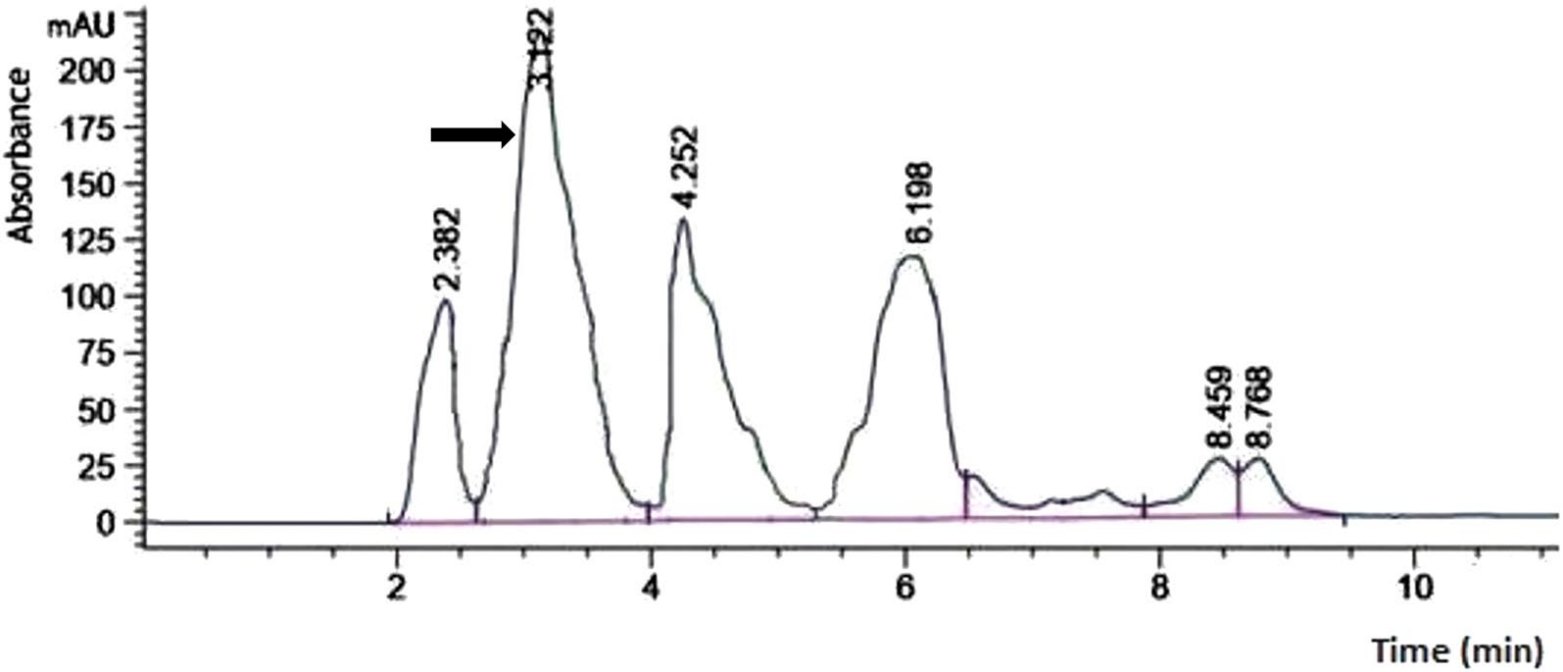
HPLC analysis profile of vitamin D _3_ bioconversion concentrated extract produced by *Actinomyces hyovaginalis* isolate A11-2 in a laboratory fermenter, showing a peak at retention time of about 3.2 min (corresponding to calcitriol) and marked by an arrow

**Fig. 2:**
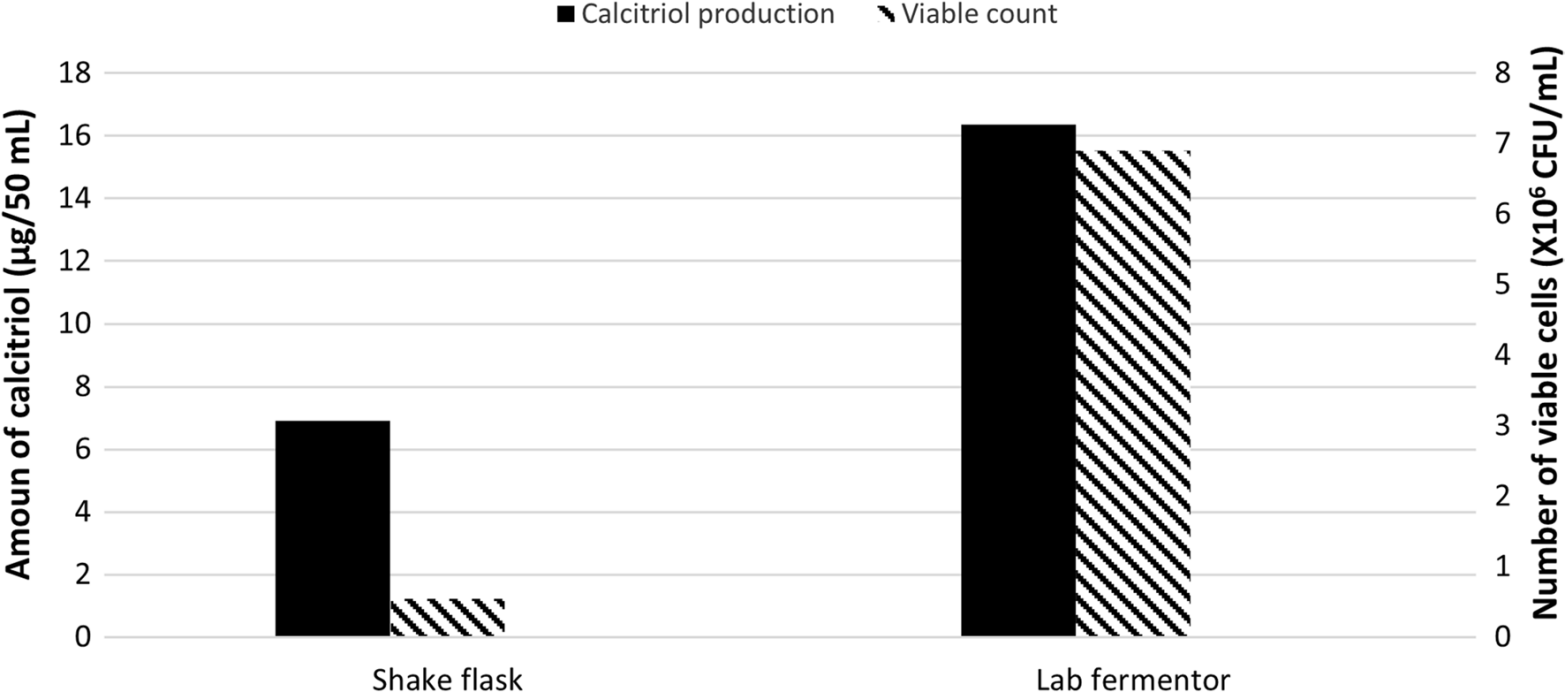
Comparison of bacterial growth and vitamin D_3_ bioconversion activity of the parent *Actinomyces hyovaginalis* isolate A11-2 in a shake flask and a laboratory fermenter (Run 1), using the same bioconversion medium. Vitamin D_3_ bioconversion activity was expressed in terms of amounts of calcitriol produced. In both cases, vitamin D_3_ (substrate) was added two days after the beginning of the main culture, and the incubation was continued for a further four days. Despite the difference between the mixing manner in the shake flask (Shaking) and that in the laboratory fermenter (Agitation), both were kept at 200 rpm. In addition, aeration was supplied by the headspace above the culture medium in case of the flask while in the fermenter, it was supplied by the introduction of sterile air at 1 vvm. Other applied culture conditions: Inoculum size of 2% v/v; uncontrolled initial pH of 7.8; the temperature of 30 °C (before substrate addition) and of 28 °C (after substrate addition)

A comparable HPLC profile for the bioconversion process was attained in the laboratory fermenter, which is in line with expectations and parallel to the study isolate’s vitamin D_3_ bioconversion activity at the shake flask level (Fig. 1). Comparisons were made between the results of the study isolate growth and its vitamin D_3_ bioconversion activity achieved in both laboratory fermenter and the shake flask (Fig. 2). Using the 14 L laboratory fermenter, the calcitriol production was increased by about 2.5-fold (32.8 µg/100 mL) to that obtained in the shake flask (12.4 µg/100 mL) as depicted in Fig. 2.

Comparison of bacterial growth and vitamin D _3_ bioconversion activity of *Actinomyces hyovaginalis* isolate A11-2 in a shake flask and a laboratory fermenter (Run 1), using the same bioconversion medium. Vitamin D _3_ bioconversion activity was expressed in terms of amounts of calcitriol produced. In both cases, vitamin D _3_ (substrate) was added two days after the beginning of the main culture, and the incubation was continued for further four days. Despite the difference between the mixing manner in the shake flask (Shaking) and that in the laboratory fermenter (Agitation), both were kept at 200 rpm. In addition, aeration was supplied by the headspace above the culture medium in the case of the flask while in the fermenter, it was supplied by the introduction of sterile air at 1 vvm. Other applied culture conditions: Inoculum size of 2% v/v; uncontrolled initial pH of 7.8; the temperature of 30 °C (before substrate addition) and of 28 °C (after substrate addition).

### Effect of different culture conditions on the fermentation process

#### Effect of aeration rate

**Fig. 3:**
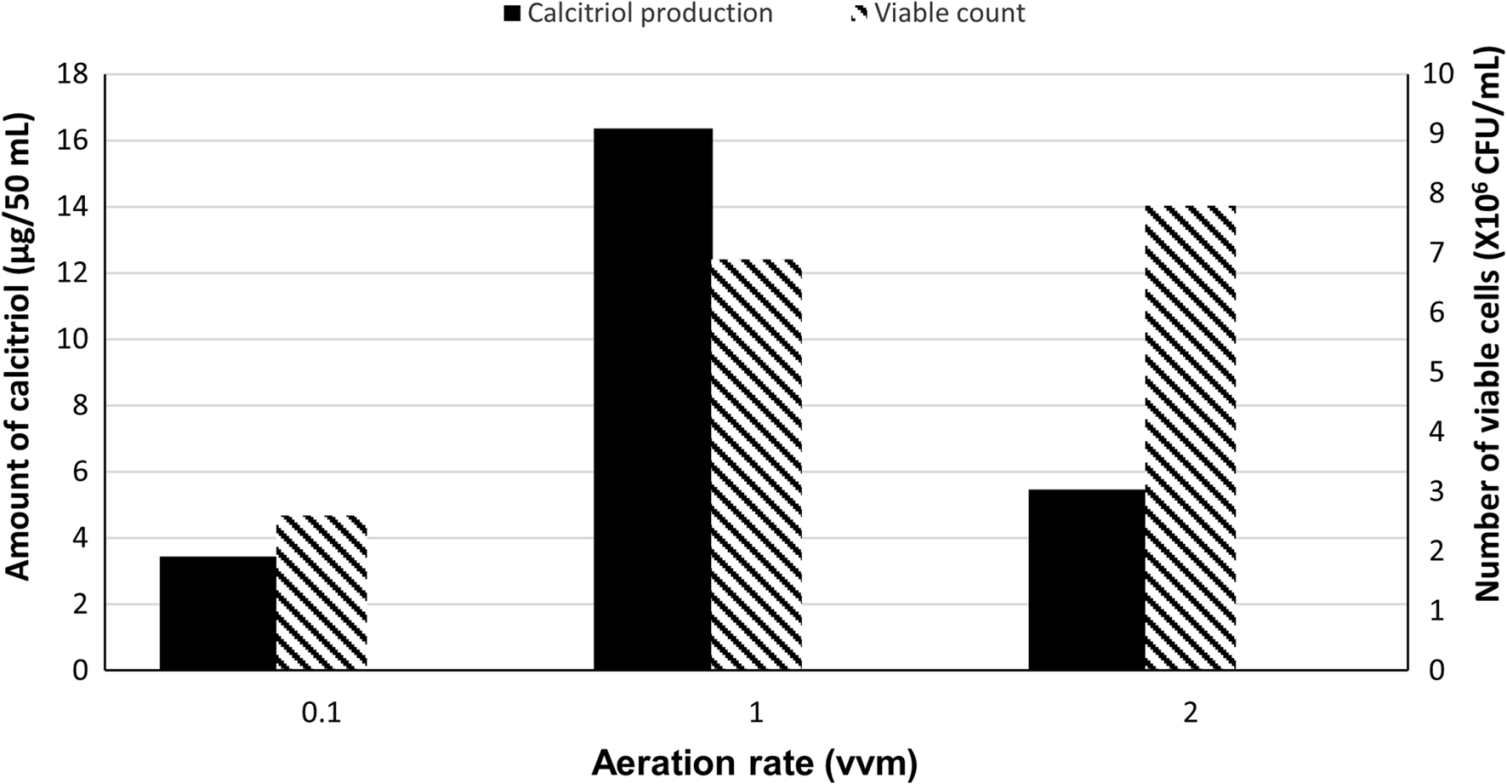
Growth and vitamin D_3_ bioconversion activity profiles of the parent *Actinomyces hyovaginalis* isolate A11-2 in a laboratory fermenter, using different aeration rates. Vitamin D_3_ bioconversion activity was expressed in terms of amounts of calcitriol produced. Other applied culture conditions: Inoculum size of 2% v/v; uncontrolled initial pH of 7.8; a temperature of 30 °C (before substrate addition) and of 28 °C (after substrate addition); agitation rate of 200 rpm; vitamin D_3_ (substrate) was added two days after the beginning of main culture

In deviation of Run 1, the bioconversion process was carried out using aeration rates of 0.1 (Run 2) vvm and 2 vvm (Run 3); one at a time. Quantification of calcitriol amounts, in vitamin D_3_ bioconversion, and concentrated extracts, was carried out using HPLC peak areas and results were compared with that obtained using an aeration rate of 1 vvm (Run 1). As illustrated in Fig. 3, The amount of the produced calcitriol using aeration rates of 0.1 (Run 2) vvm and 2 vvm (Run 3) was, 7.1 and 11.0 µg/100 mL, respectively as compared to 32.4 12.4 µg/100 mL produced in Run 1 (aeration rates of 1.0).

### Effect of agitation rate

**Fig. 4:**
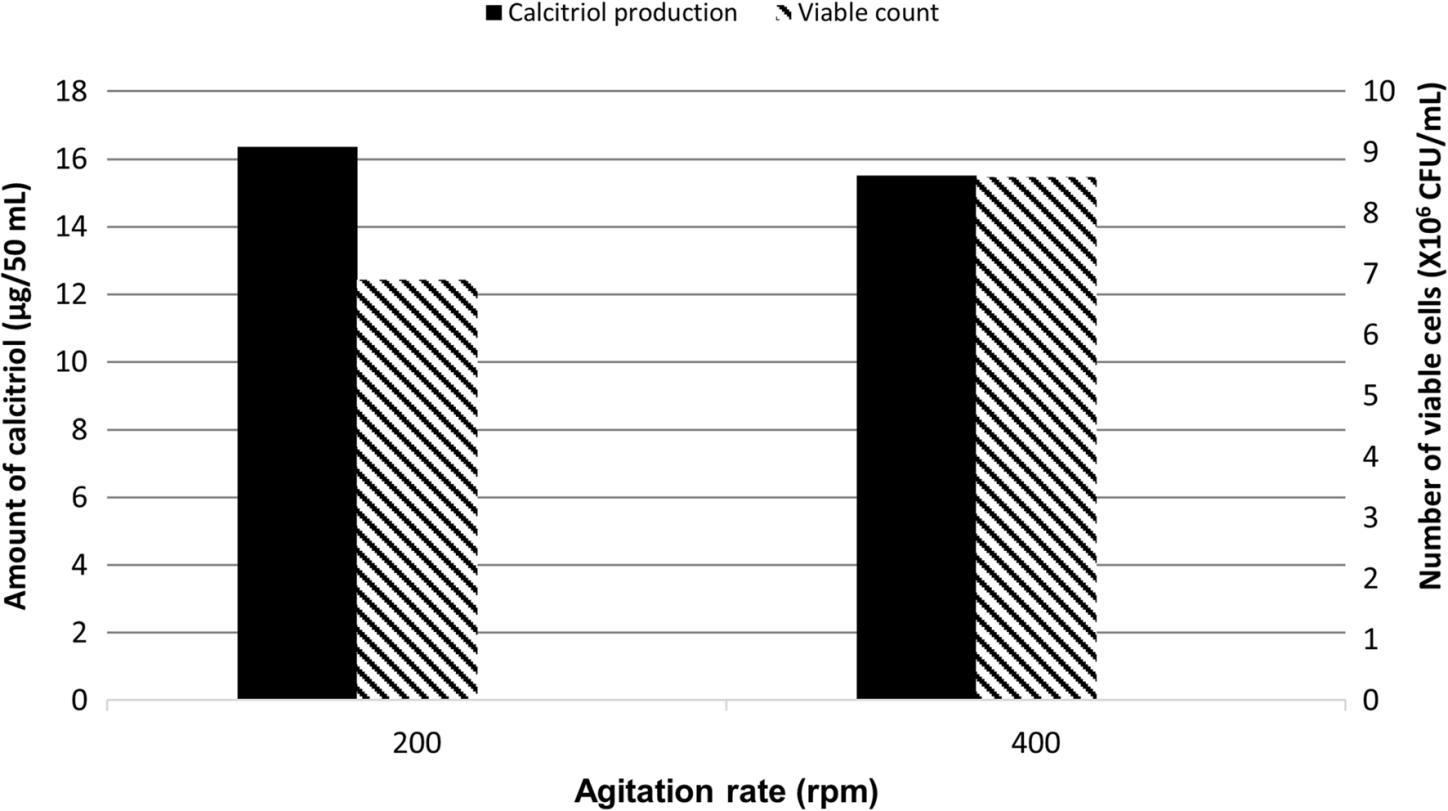
Growth and vitamin D _3_ bioconversion activity profiles of the parent *Actinomyces hyovaginalis* isolate A11-2 in a laboratory fermenter, using different agitation rates. Vitamin D _3_ bioconversion activity was expressed in terms of amounts of calcitriol produced. The aeration rate was 1 vvm, while other applied culture conditions were as those stated in the legend of Fig. 2

In deviation of Run 1 (200 rpm), the bioconversion process was carried out using an agitation rate of 400 rpm (Run 4). Quantification of calcitriol amounts, in vitamin D_3_ bioconversion concentrated extracts, was carried out using HPLC peak areas and results were compared with that obtained using agitation rate of 200 rpm (Run 1. As illustrated in Fig. 4, the amount of the produced calcitriol of Run 4 was, 30.4 µg/100 mL as compared to 32.4 µg/100 mL that produced in Run 1 (400 rpm).

### Effect of inoculum size

**Fig. 5:**
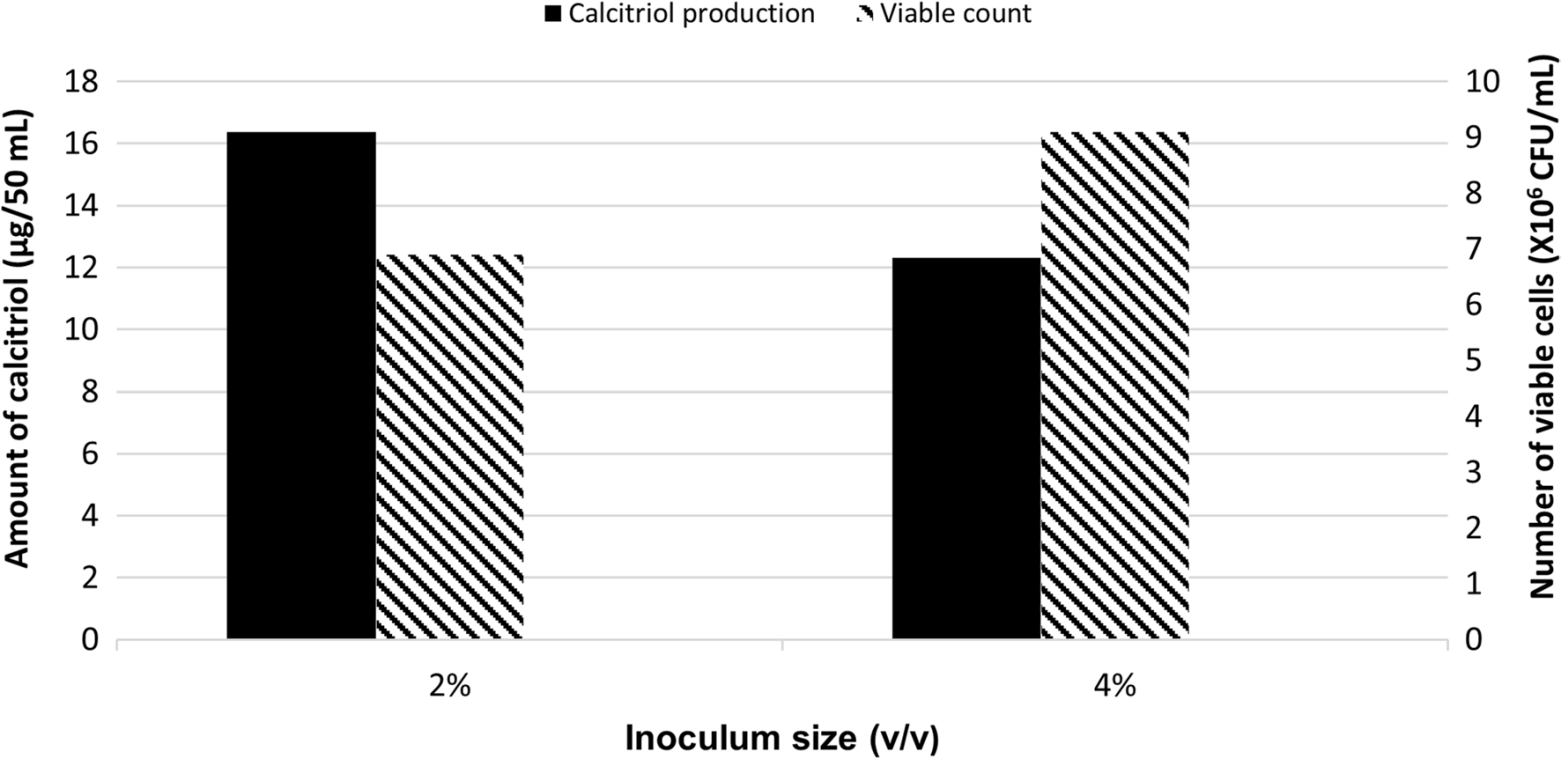
Growth and vitamin D _3_ bioconversion activity profiles of the parent *Actinomyces hyovaginalis* isolate A11-2 in a laboratory fermenter, using different inoculum sizes. Vitamin D _3_ bioconversion activity was expressed in terms of amounts of calcitriol produced. The agitation rate was 200 rpm, while other applied culture conditions were as those stated in the legend of Fig. 2

In deviation of Run 1 (2% v/v inoculum size), the bioconversion process was carried out using vegetative inoculum of 4% v/v (Run 5). Quantification of calcitriol amount, in vitamin D_3_ bioconversion concentrated extract, was conducted using HPLC peak area and the result was compared with that obtained using vegetative inoculum of 2% v/v (Run 1). As shown in Fig. 5, the amount of the produced calcitriol of Run 5 was, 24.4 µg/100 mL as compared to 32.4 µg/100 mL that produced in Run 1 (2% v/v).

### Effect of replacement of soybean with skim milk

**Fig. 6:**
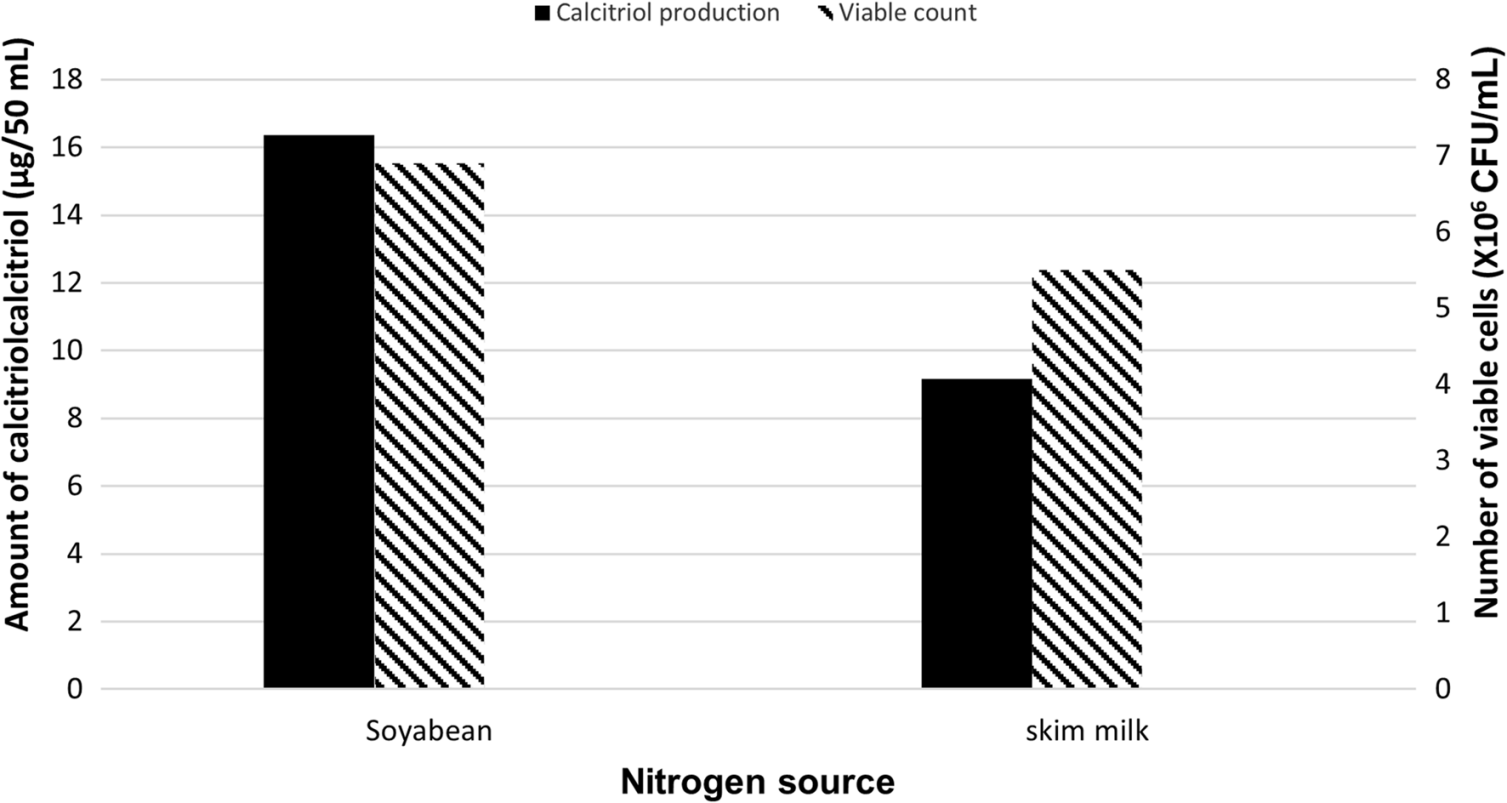
Bacterial growth and profiles of vitamin D _3_ bioconversion activity of the parent *Actinomyces hyovaginalis* isolate A11-2 in a laboratory fermenter, using soybean and skim milk as nitrogen sources. Vitamin D _3_ bioconversion activity is expressed in terms of amounts of calcitriol produced. Inoculum size was 2% v/v, while other applied culture conditions were as those stated in the legend of Fig. 2

The bioconversion process was carried out using skim milk (Run 6) instead of soybean, as the nitrogen source in the fermentation medium. Quantification of calcitriol amount, in vitamin D_3_ bioconversion concentrated extract, was carried out using HPLC peak area and the result was compared with that obtained using soybean as nitrogen source (Run 1). As depicted in Fig. 6, the amount of the produced calcitriol in Run 6 (skim milk) was, 18.2 µg/100 mL as compared to 32.4 µg/100 mL that was produced in Run 1 (soybean).

#### Effect of replacement of fructose with glucose

**Fig. 7:**
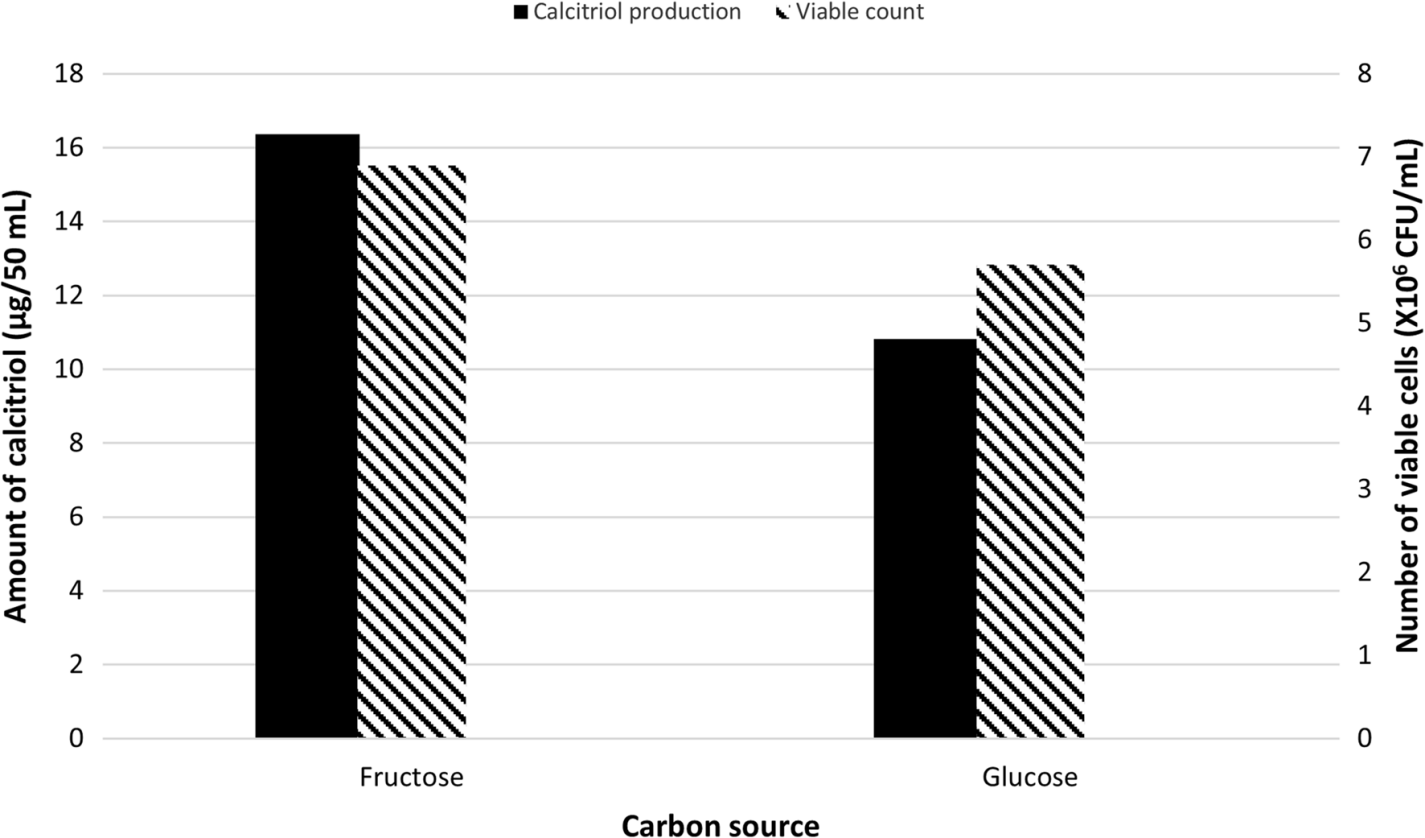
Bacterial growth and profiles of vitamin D _3_ bioconversion activity of the parent *Actinomyces hyovaginalis* isolate A11-2 in a laboratory fermenter, using fructose and glucose as carbon sources. Vitamin D _3_ bioconversion activity is expressed in terms of amounts of calcitriol produced. Soybean was used as the nitrogen source of the fermentation medium, while other applied culture conditions were as those stated in the legend of Fig. 2

The bioconversion process was carried out using glucose (Run 7) instead of fructose, as the carbon source in the fermentation medium. Quantification of calcitriol amount, in vitamin D_3_ bioconversion concentrated extract, was carried out using HPLC peak area and the result was compared with that obtained using fructose as carbon source (Run 1). As shown in Fig. 7, the amount of the produced calcitriol of Run 7 (glucose) was, 21.6 µg/100 mL as compared to 32.4 µg/100 mL produced in Run 1 (fructose).

### Effect of timing of substrate addition

**Fig. 8:**
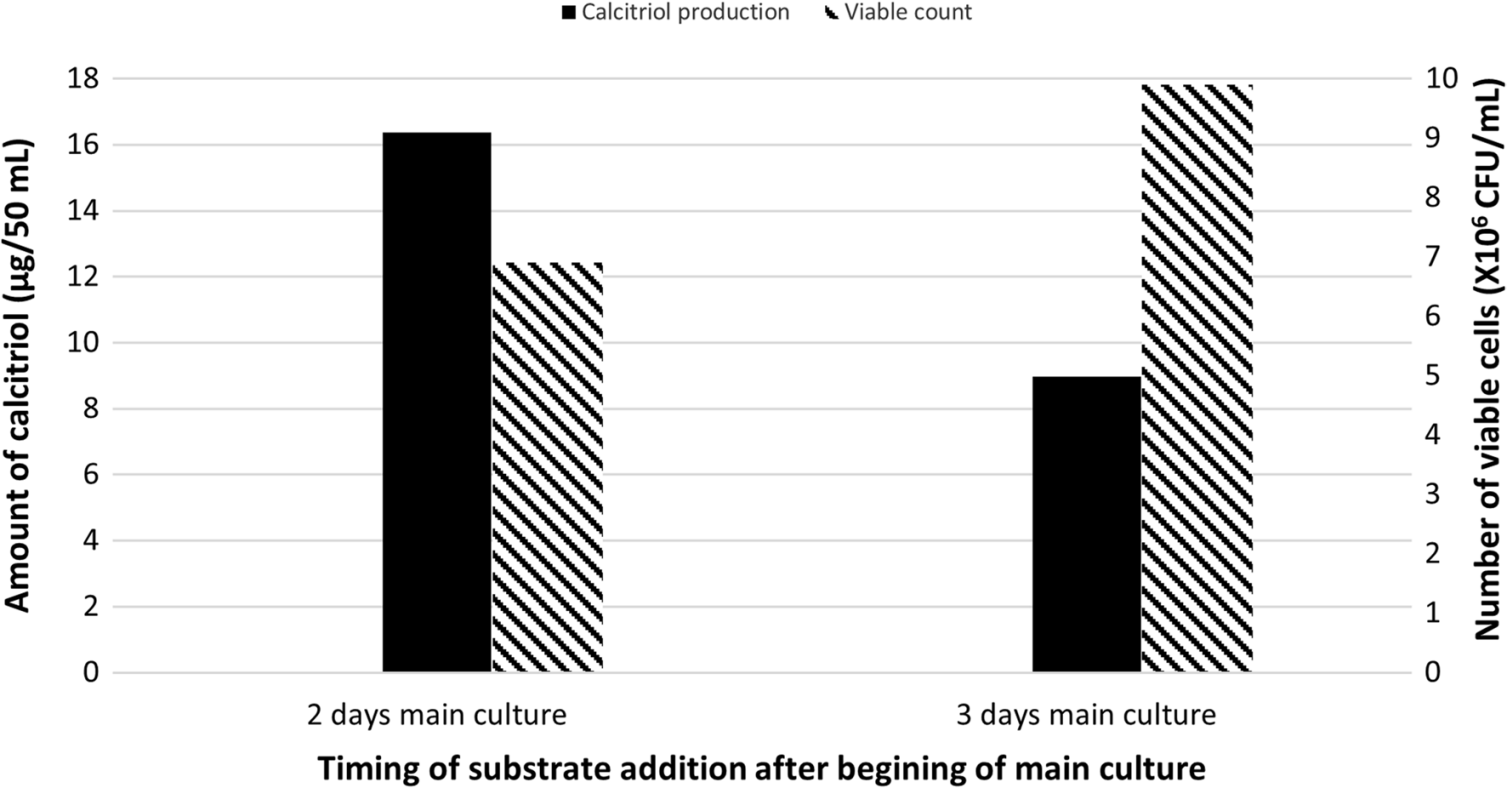
Bacterial growth and profiles of vitamin D _3_ bioconversion activity of the parent *Actinomyces hyovaginalis* isolate A11-2 in a laboratory fermenter, when adding the substrate 2 and 3 days after the beginning of main culture. Vitamin D _3_ bioconversion activity is expressed in terms of amounts of calcitriol produced. Fructose was used as the carbon source of the fermentation medium, while other applied culture conditions were as those stated in the legend of Fig. 2

The bioconversion process was carried out, as previously mentioned (materials and methods 13.1), except that the substrate (0.8 g vitamin D_3_ dissolved in 20 ml 96% ethanol) was added three days after the beginning of the main culture (Run 8). Quantification of calcitriol amount, in vitamin D_3_ bioconversion concentrated extract, was carried out using HPLC peak area and the result was compared with that obtained by the addition of the substrate two days, after main culture (Run 1), as depicted in Fig. 8. The results showed that, the amount of the produced calcitriol of Run 8 (three days after beginning of main culture) was, 17.6 µg/100 mL as compared to 32.4 µg/100 mL produced in Run 1 (two days after beginning of main culture).

#### Effect of using controlled pH

**Fig. 9:**
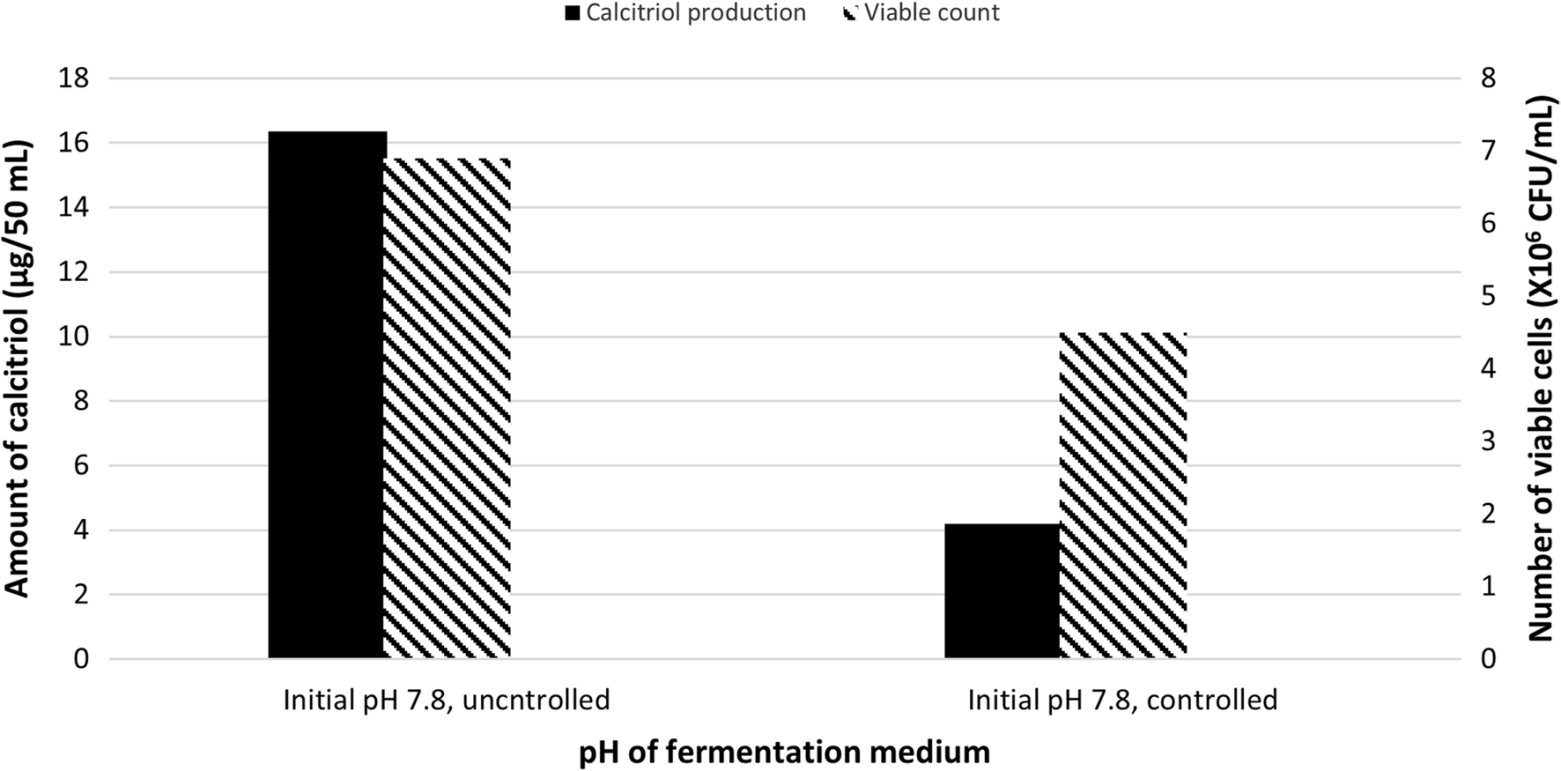
Bacterial growth and profiles of vitamin D _3_ bioconversion activity of the parent *Actinomyces hyovaginalis* isolate A11-2 in a laboratory fermenter, using controlled and uncontrolled pH values of the fermentation medium. Vitamin D _3_ bioconversion activity is expressed in terms of amounts of calcitriol produced. Vitamin D _3_ (substrate) was added two days after the beginning of the main culture, while other applied culture conditions were as those stated in the legend of Fig. 2

The bioconversion process was carried out as previously mentioned, except that pH of the medium, was fixed (controlled) at 7.8, simultaneously with substrate addition (Run 9). Quantification of calcitriol amount, in vitamin D_3_ bioconversion concentrated extract, was carried out using HPLC peak area and the result was compared with that obtained using uncontrolled pH value (Run 1), as shown in Fig. 9. The effect of different culture conditions/factors changes, mentioned before and as compared to basic fermentation process run (Run 1), on the study isolate growth and its vitamin D_3_ bioconversion activity, is summarized in Table 1.

**Table 1:** Comparison of the effects of different culture conditions/factors on growth and vitamin D_3_ bioconversion activity of *Actinomyces hyovaginalis* isolate A11-2, in a laboratory fermenter

Run #	Inoculum Size (% v/v)	Timing of substrate addition timing after main culture beginning (days)	Aeration rate (vvm)	Agitation rate (rpm)	pH control ^(a)^	C source	N source	Mean Activity^(c)^ ± S.D	Mean Growth ^(d)^ ± S.D	Specific activity ^(e)^
Run 1	2	2	1	200	Uncontrolled	Fructose	Soybean	16.4 ± 0.22	6.9 ± 0.17	47.4
Run 2	2	2	0.1	200	Uncontrolled	Fructose	Soybean	3.4 ± 0.15	2.6 ± 0.15	26.0
Run 3	2	2	2	200	Uncontrolled	Fructose	Soybean	5.5 ± 0.13	7.8 ± 0.2	14.0
Run 4	2	2	1	400	Uncontrolled	Fructose	Soybean	15.5 ± 0.22	8.6 ± 0.35	36.0
Run 5	4	2	1	200	Uncontrolled	Fructose	Soybean	12.3 ± 0.31	9.1 ± 0.17	27.0
Run 6	2	2	1	200	Uncontrolled	Fructose	Skim milk	9.2 ± 0.22	5.5 ± 0.12	33.4
Run 7	2	2	1	200	Uncontrolled	Glucose	Soybean	10.8 ± 0.15	5.7 ± 0.15	3.00
Run 8	2	3	1	200	Uncontrolled	Fructose	Soybean	8.9 ± 0.16	9.9 ± 0.32	18.0
Run 9	2	2	1	200	Controlled at 7.8 ^(b)^	Fructose	Soybean	4.2 ± 0.32	4.5 ± 0.12	18.0

S.D. (standard deviation)

^a^Initial pH was 7.8

b pH control was done at the same time of substrate addition

^c^ Expressed in terms of the amount of calcitriol produced (µg/50 mL)

^d^Expressed in terms of viable count (X 10^6^ CFU/mL)

^e^Expressed in terms of the amount of calcitriol produced (ng) per 1 million CFU

## Discussion

Several prior research studies have applied fermenters of various sizes to study the microbial conversion of vitamin D_3_ into its hydroxylated derivatives (Kang et al. 2006, 2015a, b; Abass et al. 2011a, b; Luo et al. 2016; Tang et al. 2020; Wang et al. 2022). A previous study conducted by Sasaki et al. 1991, where 300 *Streptomyces* isolates were screened, found two strains with a conversion productivity of about 7.0 mg/L/min. In subsequent studies, genetically engineered strains (Ehrhardt et al. 2016) were used to boost 25(OH)VD3 production. Nisin-treated *Rhodococcus* cells containing VdhT107A could increase 25(OH)VD3 production to 573 mg/L in just 2 h (Yasutake et al. 2013). In addition, Luo et al. 2017 tested *Pseudonocardia autotrophica* and was found to produce the highest 25(OH)VD3 concentration (639 mg/L) within 120 h. Interestingly, some studies have examined the vitamin D _3_ bioconversion capability of the new strain called *Sterolibacterium denitrificans* and were reported with highly regioselective hydroxylation of VD3, with the highest 25(OH)VD3 concentration of 1.4 g/L and by using the crude enzyme preparation (Rugor et al. 2017; Szaleniec et al. 2018; Warnke et al. 2016).

Despite the rising number of research on vitamin D_3_ bioconversion into calcitriol, further studies are still required that can significantly contribute to the improvement of such a bioconversion process. A previous study was conducted in our Lab to optimize culture conditions for the transformation of vitamin D_3_ to calcitriol by *Actinomyces hyovaginalis* CCASU-A11-2 in a shake flask Abass et al. 2011b). Comparison of bacterial growth and vitamin D _3_ bioconversion activity of *Actinomyces hyovaginalis* isolate A11-2 in a shake flask and a laboratory fermenter, using the same bioconversion medium. Vitamin D _3_ bioconversion activity was expressed in terms of amounts of calcitriol produced. In both cases, vitamin D _3_ (substrate) was added two days after the beginning of the main culture, and the incubation was continued for further four days. Despite the difference between the mixing manner in the shake flask (Shaking) and that in the laboratory fermenter (Agitation), both were kept at 200 rpm. In addition, aeration was supplied by the headspace above the culture medium in the case of the flask while in the fermenter, it was supplied by the introduction of sterile air at 1 vvm. Other applied culture conditions: Inoculum size of 2% v/v; uncontrolled initial pH of 7.8; the temperature of 30 °C (before substrate addition) and of 28 °C (after substrate addition).

Therefore, this work aimed to improve such bioconversion process, in a 14 L laboratory fermenter. From this perspective, a batch fermentation mode in a 14 L laboratory fermenter was used to test the respective isolate’s ability to convert vitamin D _3_ into calcitriol. Accordingly, applied fermentation conditions (Run1) were established and these included: basal medium used for main culture and vitamin D_3_ bioconversion; inoculum size of 2% v/v; agitation rate of 200 rpm; aeration rate of 1 vvm; initial pH of 7.8 (uncontrolled); temperature of 30 °C (before substrate addition) and of 28 °C (after substrate addition); addition of vitamin D_3_ two days after the beginning of main culture and continual of the incubation process for additional four days. It is crucial to note that several of the conditions/factors previously described were based on a prior screening effort for optimizing vitamin D_3_ bioconversion, by the research isolate, in a shake flask (Abass et al. 2011b; Wang et al. 2022; ).

Similar behavior was observed when the study isolate’s bacterial growth and vitamin D_3_ bioconversion activity profiles were compared in the fermenter (Run 1) and shaking flask. However, the maximum values for the two evaluated parameters were higher in the fermenter, both after four days of fermentation and under the same culture conditions. It is worth saying that both the shake flask and the fermenter used different techniques to reach the same agitation rate of 200 rpm: orbital shaking in the flask and mechanical stirring in the fermenter. Additionally, in a shake flask, aeration was provided by the headspace above the culture medium and shaking, whereas in a fermenter, aeration was provided by the introduction of sterile air at 1 vvm and stirring. Under the applied conditions, the isolate of our study in the laboratory fermenter produced around 16.4 µg/50 mL of calcitriol from vitamin D_3_, and the biomass was about 6.9 × 10^6^ CFU/mL (Run 1). Our results showed that, using the 14 L laboratory fermenter, the calcitriol production was increased by about 2.5-fold with a conversion yield 32.8 µg/100 mL (6.9 × 10^6^ CFU/mL) to that obtained in the shake flask of 12.4 µg/100 mL (1.0 × 10^6^ CFU/mL). It is widely known that the composition of the medium, the culture process, and the physiologic factors such as pH, aeration rate, and agitation rate, affect the rate of conversion of compounds with microbial enzymes or intact cells **(**Kulprecha et al. 1985; Sasaki et al. 1992; Flores et al. 1997; Uzura et al. 2001; Sawada et al. 2004; Tang et al. 2020). As a result, in the current study, various culture conditions/factors that might affect the study isolate’s ability to bioconvert vitamin D_3_ were investigated. The condition/factor value that seemed to be best for the generation of calcitriol was applied in the following experiments.

As shown in our results, employing aeration rates of either 0.1 (Run 2) or 2 vvm (Run 3) caused the study isolate’s bioconversion activity to diminish (by around 80 and 65%, respectively), in comparison to that induced by using aeration rates of 1 vvm (Run 1). The abrupt decrease for calcitriol, produced at aeration rate of 0.1 vvm, may be largely correlated to the decrease in biomass caused by such aeration rate, and to a lesser extent to the impact of the low aeration rate on the bioconversion process. On the other hand, greater biomass was obtained with a 2 vvm aeration rate. Nevertheless, calcitriol synthesis was significantly lower than that at 1 vvm of aeration, primarily due to the high aeration rate’s oxidative destruction of the synthesized calcitriol (Raiteri et al. 2021). As displayed in our results, utilizing an agitation rate of 400 rpm (Run 4) resulted in a drop (by around 5%) in the study isolate’s bioconversion activity in comparison to that produced by an agitation rate of 200 rpm (Run 1). Despite producing more biomass, calcitriol production decreased at 400 rpm, most likely because of oxidative destruction of the produced calcitriol brought on by the high agitation rate. It should be noted that attempts at an agitation rate of 600 rpm were also made, but unfortunately, the runs were not successful due to enormous, uncontrollable froth, which in turn produced major leakage of the fermentation medium’s working volume.

As depicted in the results, using an inoculum size of 4% v/v (Run 5), caused a decline (by about 25%) in the bioconversion activity of the study isolate, as compared to that caused at an inoculum size of 2% v/v (Run 1). Despite resulting in higher biomass, the results revealed the absence of coherency between the effect of increasing the inoculum size on the study isolate growth and its effect on calcitriol production. As mentioned before, soybean was employed as the nitrogen source in the basal medium, used for the main culture and vitamin D_3_ bioconversion process. In our work, an experiment was carried out utilizing skim milk (Run 6) instead of soybean. The decision to use skim milk was based on a prior study conducted in a shake flask which depicted that, of many nitrogen sources tested, skim milk and soybean were the best for the study isolate’s bioconversion ability (Abass et al. 2011b). The bioconversion activity of the tested isolate decreased (by around 45%), when skim milk was used. This result differs from what was discovered in Kang’s work (Kang et al. 2006) to optimize vitamin D_3_ bioconversion into calcitriol. The decreased biomass produced by using skim milk is the most likely cause of the decline in calcitriol production. Similar to this, fructose was used as the carbon source in the basal medium, used for the main culture and vitamin D_3_ bioconversion process. In an experiment, glucose was employed in place of fructose (Run 7). The choice of glucose was based on a prior study conducted in a shake flask which showed that of the many carbon sources evaluated, fructose and glucose were the best for the study isolate’s ability to bioconvert vitamin D_3_ (Abass et al. 2011b). As shown in our results, using glucose decreased the bioconversion activity by about 35%. This result differs from what was concluded in Kang’s work for the optimization of vitamin D_3_ bioconversion (Kang et al. 2006). The decrease in calcitriol production is most likely caused by the reduced biomass caused by using glucose.

As shown in our results regarding the timing of substrate addition, adding vitamin D_3_ two days after the beginning of the main culture (Run 1) was found to be the better timing to produce calcitriol. Regarding growth, higher biomass was obtained when vitamin D_3_ was added three days after the beginning of the main culture. However, a decrease in the amount of calcitriol produced (by about 45%), occurred four days after the addition of vitamin D3 (same conversion time). In the experiment done for investigating the effect of using controlled pH, pH of the fermentation medium, was fixed (controlled) at 7.8, simultaneously with substrate addition (Run 9). This resulted in about 75% decrease in the amount of calcitriol produced. Accordingly, our results revealed that the uncontrolled pH (initial pH was fixed to 7.8 at the beginning of the fermentation process) was more favorable for calcitriol production as compared to the controlled pH experiment (Run 9). The cause of that is the decrease in biomass brought on by the fixing of pH value. It is significant to note that, calcitriol production was generally found to be correlated with the study isolate biomass for the tested condition/factor, except for the following: (1) Conditions that made the product more susceptible to oxidative degradation, such as employing 2 vvm aeration rate or 400 rpm for agitation; (2) Conditions that resulted in high biomass at the time of substrate addition, including employing an inoculum size of 4% v/v or adding vitamin D_3_ three days after the primary culture started. Finally, and as inferred from Table 1, the specific vitamin D_3_ bioconversion activity of the studied isolate, as measured by the quantity of calcitriol produced per one million bacterial CFU, reduced, as compared to basic fermentation run (Run 1), by about: (1) 45% by using aeration rate of 0.1 vvm; (2) 70% by using aeration rate of 2 vvm; (3) 25% by using agitation rate of 400 rpm; (4) 4–43% by using inoculum size of 4% v/v; (5) 5–30% by using skim milk as the fermentation medium nitrogen source; (6) 6–20% by using glucose as the fermentation medium carbon source; and 7) 62% by either adding the substrate (vitamin D_3_) three days after main culture beginning or fixing the pH value of the fermentation medium at 7.8. Accordingly, the aeration rate (Bligh and Dyer 1959), inoculum size, the timing of substrate addition, and fixed pH of the fermentation medium are the most important variables that appeared to affect the study isolate’s ability to bioconvert vitamin D_3_. Therefore, for industrial applications, such factors should be carefully considered. Inoculum size of 2% v/v, agitation rate of 200 rpm, aeration rate of 1 vvm, initial pH of 7.8 (uncontrolled), the addition of vitamin D_3_ 2 days after the beginning of main culture, and use of fructose as the medium carbon source and defatted soybean as the nitrogen source were found to be the ideal bioconversion conditions.

## Supplementary Information

**Additional file 1: TableS1.** List of the different conditions of thenine fermentation runs (run-1-run 9).

## Data Availability

All data generated or analyzed during this study are included in this published article and supplementary file.
