# Supplementary material for: Bioconversion of vitamin D3 into calcitriol by Actinomyces hyovaginalis isolate CCASU- A11-2
Source: AMB Express. 2023 Jul 12;13:73. doi: 10.1186/s13568-023-01574-3 (PMC10335987; doi:10.1186/s13568-023-01574-3)
Supplement: Supplementary file 1 — Additional file 1: TableS1. List of the different conditions of thenine fermentation runs (run-1-run 9). [file 13568_2023_1574_MOESM1_ESM.docx]

**Table S1**. List of the different conditions of the nine fermentation runs (run-1-run 9)

| **Run #** | **Inoculum Size (% v/v)** | **Timing of substrate addition timing after main culture beginning (days)** | **Aeration rate (vvm)** | **Agitation rate (rpm)** | **pH control** ^(a)^ | **C source** | **N source** |
| --- | --- | --- | --- | --- | --- | --- | --- |
| Run 1 | 2 | 2 | 1 | 200 | Uncontrolled | Fructose | Soybean |
| Run 2 | 2 | 2 | 0.1 | 200 | Uncontrolled | Fructose | Soybean |
| Run 3 | 2 | 2 | 2 | 200 | Uncontrolled | Fructose | Soybean |
| Run 4 | 2 | 2 | 1 | 400 | Uncontrolled | Fructose | Soybean |
| Run 5 | 4 | 2 | 1 | 200 | Uncontrolled | Fructose | Soybean |
| Run 6 | 2 | 2 | 1 | 200 | Uncontrolled | Fructose | Skim milk |
| Run 7 | 2 | 2 | 1 | 200 | Uncontrolled | Glucose | Soybean |
| Run 8 | 2 | 3 | 1 | 200 | Uncontrolled | Fructose | Soybean |
| Run 9 | 2 | 2 | 1 | 200 | Controlled at 7.8 ^(b)^ | Fructose | Soybean |
